# Tacaribe Virus but Not Junin Virus Infection Induces Cytokine Release from Primary Human Monocytes and Macrophages

**DOI:** 10.1371/journal.pntd.0001137

**Published:** 2011-05-10

**Authors:** Allison Groseth, Thomas Hoenen, Michaela Weber, Svenja Wolff, Astrid Herwig, Andreas Kaufmann, Stephan Becker

**Affiliations:** 1 Institut für Virologie, Philipps Universität Marburg, Marburg, Germany; 2 Institut für Immunologie, Philipps Universität Marburg, Marburg, Germany; University of Texas Medical Branch at Galveston, United States of America

## Abstract

The mechanisms underlying the development of disease during arenavirus infection are poorly understood. However, common to all hemorrhagic fever diseases is the involvement of macrophages as primary target cells, suggesting that the immune response in these cells may be of paramount importance during infection. Thus, in order to identify features of the immune response that contribute to arenavirus pathogenesis, we have examined the growth kinetics and cytokine profiles of two closely related New World arenaviruses, the apathogenic Tacaribe virus (TCRV) and the hemorrhagic fever-causing Junin virus (JUNV), in primary human monocytes and macrophages. Both viruses grew robustly in VeroE6 cells; however, TCRV titres were decreased by approximately 10 fold compared to JUNV in both monocytes and macrophages. Infection of both monocytes and macrophages with TCRV also resulted in the release of high levels of IL-6, IL-10 and TNF-α, while levels of IFN-α, IFN-β and IL-12 were not affected. However, we could show that the presence of these cytokines had no direct effect on growth of either TCRV of JUNV in macrophages. Further analysis also showed that while the production of IL-6 and IL-10 are dependent on viral replication, production of TNF-α also occurs after exposure to UV-inactivated TCRV particles and is thus independent of productive virus infection. Surprisingly, JUNV infection did not have an effect on any of the cytokines examined indicating that, in contrast to other viral hemorrhagic fever viruses, macrophage-derived cytokine production is unlikely to play an active role in contributing to the cytokine dysregulation observed in JUNV infected patients. Rather, these results suggest that an early, controlled immune response by infected macrophages may be critical for the successful control of infection of apathogenic viruses and prevention of subsequent disease, including systemic cytokine dysregulation.

## Author Summary

It remains unclear how arenavirus infection causes disease; however, for other hemorrhagic fever viruses, infection has been linked to over-production of numerous cytokines by macrophages that can then affect vascular integrity. In order to determine if a similar mechanism might contribute to arenavirus pathogenesis, we have examined the infection and subsequent cytokine production in human monocytes and macrophages by two closely related arenaviruses: the apathogenic Tacaribe virus (TCRV) and the hemorrhagic fever-causing Junin virus (JUNV). We found that both viruses infected primary monocyte and macrophage cultures; however, only, in the case of TCRV was infection accompanied by the production of cytokines. These cytokines would have the potential to stimulate an antiviral response to infection, including the production of antibodies, which are known to be protective during infection. Surprisingly, in contrast to what is observed in other viral hemorrhagic fevers, we found that JUNV infection did not have any effect on the expression of these cytokines. This suggests that an early, strong immune response by infected macrophages may be critical for the control of infection by apathogenic arenaviruses and the prevention of subsequent disease.

## Introduction

The *Arenaviridae* constitute a large family of bi-segmented single-stranded RNA viruses, with 25 species currently being recognized [1], [2], [3], [4] and new members being discovered approximately once every 3 years [5]. The arenaviruses include several significant human pathogens capable of causing hemorrhagic fever (HF), and as such they represent a significant threat to human health worldwide. In particular, members of the New World arenavirus serocomplex are responsible for at least 5 distinct HF diseases, collectively termed the South American hemorrhagic fevers, which are associated with a combination of hemorrhagic and neurologic symptoms [2], [6]. Of these, the most clinically significant in terms of disease burden is Junin virus (JUNV), which is the causative agent of Argentine hemorrhagic fever (AHF). Since it was first identified in the 1950s JUNV infection has typically been responsible for 300–1000 cases of AHF per year. However, since the early 1990s case numbers of AHF have dropped dramatically due to an effective targeted vaccination program in the endemic region [7], [8]. Infection is most commonly seen among agricultural workers and occurs seasonally in the humid pampas of the provinces of Buenos Aires, Santa Fe, Córdoba, and La Pampa in Argentina, with infection believed to occur through cutaneous or mucosal contact to contaminated blood, feces or urine from infected rodents, often in the form of aerosols [9]. The majority of these infections with JUNV result in clinical disease [9], which can be divided into three distinct phases: prodromal, neurological–hemorrhagic, and convalescent [7]. The prodromal phase occurs during the first week after onset of symptoms and can include non-specific symptoms such as fever (38–39°C), malaise and myalgia as well as petechiae on the soft palate and cutaneous petechiae [9]. Subsequently, 20–30% of AHF cases progress to the neurologic–hemorrhagic phase of disease characterized by severe hemorrhagic or neurological manifestations and shock [9].

Despite being responsible for a growing number of hemorrhagic fevers, the pathophysiological mechanisms underlying the development of disease, and particularly hemorrhagic disease, during arenavirus infection are poorly understood. However, the involvement of elevated cytokines levels in the pathophysiology of AHF is suggested by descriptive studies of infected patients where elevated levels of interferon (IFN)-α, interleukin (IL)-6, IL-8, IL-10 and tumour necrosis factor (TNF)-α have been reported [10], [11], [12]. Further, the levels of these cytokines have been shown to correlate with disease severity, strongly suggesting a role in pathogenesis [10], [11], [12]. Thus it seems possible that some of the processes related to the development of hemorrhagic fever in JUNV patients may parallel those known to occur in better characterized viral hemorrhagic fevers, such as those caused by filoviruses. However, despite evidence for a role in the pathophysiology of Argentine hemorrhagic fever, data regarding the source of the cytokines detected in patients during infection is presently lacking. As with many other hemorrhagic fever viruses, infection of macrophages as a primary target cell type is believed to play a crucial role in the establishment of arenavirus infections [13], [14], [15], making them a promising candidate as the source of these elevated cytokine levels. Indeed, for filoviruses it has been shown that this infection triggers the secretion of high levels of pro-inflammatory cytokines, particularly TNF-α [16], [17], which then cause changes in the permeability of the vascular endothelium that lead to the clinical manifestations of hemorrhage and shock [16].

In addition to New World arenaviruses causing South American hemorrhagic fever, a number of human apathogenic New World arenaviruses have also been identified. Interestingly, in some cases these viruses are closely related to HF-causing arenaviruses. For instance, Tacaribe virus (TCRV) is closely related to JUNV, however, despite over 60 years of research on this virus only a single case of febrile disease has ever been reported [18], [19], [20]. The close phylogenetic relationships between such pairs of virulent and avirulent family members provide an excellent opportunity to identify critical differences in pathogenesis related processes. Using this approach, we have compared the effects of TCRV and JUNV infection on human monocytes and macrophages, with a particular emphasis on the production of cytokines known to be associated with AHF infection, in order to determine what role the production of macrophage-derived cytokines in response to virus infection might be playing in arenavirus pathogenesis.

We found that, despite showing robust growth in VeroE6 cells, TCRV replication is reduced in primary human monocytes and macrophages, in comparison to JUNV, and is accompanied by significant up-regulation of IL-6, IL-10 and TNF-α. However, these cytokines do not appear to be directly responsible for the diminished virus titres in these cells. Further, IL-6 and IL-10 production were shown to be dependent on productive infection, while TNF-α production was not. In contrast to TCRV, JUNV infection did not stimulate production of any of the cytokines examined. Thus these observations support a model of arenavirus pathogenesis in which early cytokine production as a result of macrophage infection may actually be protective.

## Materials and Methods

### Viruses and continuous cell lines

Tacaribe virus and Junin virus (strain Romero) were kindly provided by Dan Kolakofsky (University of Geneva) and Heinz Feldmann (Public Health Agency of Canada), respectively, and stocks were prepared as previously described [21]. Vesicular stomatis virus (VSV) was provided by Friedemann Weber (Philipps Universität Marburg) and Sendai virus (SeV) was provided by Marianne Nain (Philipps Universität Marburg). All work with JUNV was carried out in the BSL-4 laboratory at the Philipps Universität Marburg.

VeroE6 (African green monkey kidney) cells were maintained in Dulbecco's modified Eagle's medium (DMEM, Invitrogen) supplemented with 10% fetal bovine serum (FBS, PAN Biotech), 2 mM L-glutamine (Invitrogen), 100 U/ml penicillin and 100 µg/ml streptomycin (Invitrogen) and grown at 37°C and 5% CO_2_.

### Isolation and culture of primary human monocytes and macrophages

Leukocyte-enriched buffy coats from healthy anonymous donors were obtained from the blood bank of the Marburg-Gießen University Hospital. Samples were layered on 15 ml of Lymphocyte Separation Medium (1.77 g/ml; PAA Laboratories) in a 50 ml tube. The blood was centrifuged at 600×g for 30 min at room temperature (RT) with no brake. The layer formed by the mononuclear cells was carefully removed and washed twice with 50 ml of ice-cold PBS supplemented with magnesium and calcium (+Mg/+Ca). After each wash step the cells were spun down at 300×g for 7 min at 4°C. After the second centrifugation, the cells were resuspended in RPMI-1640 with 2% human AB serum (Sigma) at a concentration of 1.5×10^6^ cells/ml, and 4 ml per well was seeded into Primaria 6-well plates (BD Biosciences) and incubated at 37°C and 5% CO_2_. After one hour, the medium was removed and the cells were thoroughly washed three times with PBS (+Mg/+Ca) to remove the lymphocytes. Then 4 ml of RPMI-1640 with 5% human AB serum was added to the cells, and they were incubated for 1 day (monocytes) or 7 days (macrophages) at 37°C and 5% CO_2_ before use.

### Virus infection of VeroE6 cells

VeroE6 cell monolayers with a confluence of 80–90% were infected in 6-well plates with TCRV or JUNV at an MOI of 1 or 0.1 in 1 ml of serum-free DMEM for 60 min at 37°C in a 5% CO_2_ atmosphere. Following absorption the inoculum was removed and the cells washed 3x with DMEM to remove any unbound virus. Cells were placed in fresh DMEM containing 2% FBS (PAN Biotech), 2 mM L-glutamine (Invitrogen), 100 U/ml penicillin and 100 µg/ml streptomycin (Invitrogen) and incubated for 5 days. Samples from the supernatant were collected every 24 h for analysis of progeny virus release by plaque assay.

### Virus infection of primary human monocytes and macrophages

Monocyte or macrophage cultures generated as described above were infected in 6-well Primaria (BD Biosciences) plates with TCRV or JUNV at an MOI of 0.1 or mock infected, in 1 ml of serum-free RPMI-1640 for 60 min at 37°C in a 5% CO_2_ atmosphere. Following absorption the inoculum was removed and the cells washed 3x with RPMI-1640 to remove any unbound virus. Cells were placed in fresh RPMI-1640 containing 2% human AB serum (Sigma), 2 mM L-glutamine (Invitrogen), 100 U/ml penicillin and 100 µg/ml streptomycin (Invitrogen) and incubated for 4 days. Samples were collected daily for analysis of progeny virus release by plaque assay and after 4 days for cytokine release. As positive controls for IFN-α, IFN- β and IL-12 production, monocyte and macrophage cultures were infected with 64 or 640 HAU of SeV per well, or stimulated with lipopolysaccharide (LPS, Sigma) at a concentration of 100 ng/mL or 1 µg/ml. All samples infected with SeV or stimulated with LPS were harvested after 20 h for analysis of cytokine release.

### Plaque assay for titre determination

VeroE6 cell monolayers with a confluence of 80–90% were infected in a 6- or 12-well plate format with ten-fold dilution series of samples containing TCRV or JUNV for 60 min at 37°C in a 5% CO_2_ atmosphere in serum-free DMEM. For 12-well plates an infection volume of 250 µl was used, while for 6-well plates an infection volume of 500 µl was used. Plates were rocked every 15 minutes during infection. Following absorption the inoculum was removed and the monolayers were then overlaid with 2 ml (12 well) or 4 ml (6 well) of Minimum Essential Medium (MEM) containing BactoAgar (BD Biosciences) at a final concentration of either 0.7% (TCRV) or 0.9% (JUNV), in addition to 2% FBS, 2 mM L-glutamine (Invitrogen), 100 U/ml penicillin and 100 µg/ml streptomycin (Invitrogen). Plaques were allowed to develop for either 5–6 days (JUNV) or 7–8 days (TCRV) before being fixed and stained with a 0.1% crystal violet solution (10% formaldehyde, 0.1% crystal violet).

### Cytokine ELISAs

The presence of IFN-α and IFN-β (Pestka Biomedical Laboratories), as well as IL-6, IL-10, IL-12 and TNF-α (R&D Systems) were detected using commercial assays according to the manufacturer's directions. Monocyte and macrophage supernatants were analysed undiluted Due to the high signals obtained, analysis of TCRV samples for IL-6, IL-10 and TNF-α was also carried out using samples diluted 1∶10 in the sample dilution buffers provided by the manufacturer.

### Interferon bioassay

In order to further analyze the IFN content of supernatants derived from infected monocytes and macrophages, these supernatants, or known concentrations of recombinant human IFN-β (Calbiochem) were incubated on 80% confluent VeroE6 cells for 18 h. Cells were then washed once with DMEM and infected with vesicular stomatitis virus (VSV) at an MOI of 0.001 for 1 h at 37°C. Following infection the inoculum was removed and fresh DMEM containing 2% FBS (PAN Biotech), 2 mM L-glutamine (Invitrogen), 100 U/ml penicillin and 100 µg/ml streptomycin (Invitrogen) was added. Cells were then incubated overnight at 37°C with monolayers examined for the extent of CPE formation after 12, 18 and 24 h.

### Inactivation of arenaviruses by UV irradiation

Stock preparations of TCRV or JUNV with a known starting titre of 1×10^6^ pfu/ml (JUNV) or 2×10^6^ pfu/ml (TCRV) were inactivated as previously described [22], [23] by irradiation at 254 nm using a UV Lamp (CAMAG) for 1 h. Samples of virus stocks inactivated using this method were analyzed by plaque assay as described above, to ensure complete inactivation (data not shown).

### Removal of virus from cell culture supernatants by ultracentrifugation

To generate virus-free cytokine-containing supernatants, 1 ml aliquots of supernatant from TCRV, JUNV or Mock infected monocytes were loaded into 1.5 ml polyallomer microfuge tubes (Beckmann) and centrifuged at 55,000 rpm in a TLA-55 rotor (186,000×g; Beckmann) for 2.5 h at 4°C. Subsequently 900 ul from each aliquot was transferred to a fresh tube and centrifuged again under the same conditions. Finally 800 ul was transferred to a fresh tube. Clarified supernatants were examined by plaque assay to confirm the removal of all infectious virus (data not shown).

### Treatment of macrophages with monocyte culture supernatants

Cytokine-containing supernatants, derived from infected monocyte cultures from which virus had been removed, were applied to new macrophages cultures prepared in 6 well plates as described above. Cells were then incubated at 37°C with the relevant supernatant for 2 h prior to infection. Stock virus with a known titre was then added to the supernatant to generate an MOI of 0.1 per cell. Cells were then incubated for an additional 1 h at 37°C. Following absorption the inoculum was removed and fresh RPMI-1640 containing 2% human AB serum (Sigma), 2 mM L-glutamine (Invitrogen), 100 U/ml penicillin and 100 µg/ml streptomycin (Invitrogen) was added. Supernatants were harvested after 4 days for analysis of virus growth.

## Results

### Junin and Tacaribe replicate with comparable efficiency in VeroE6 cells, but not in primary monocytes and macrophages

**Figure 1:**
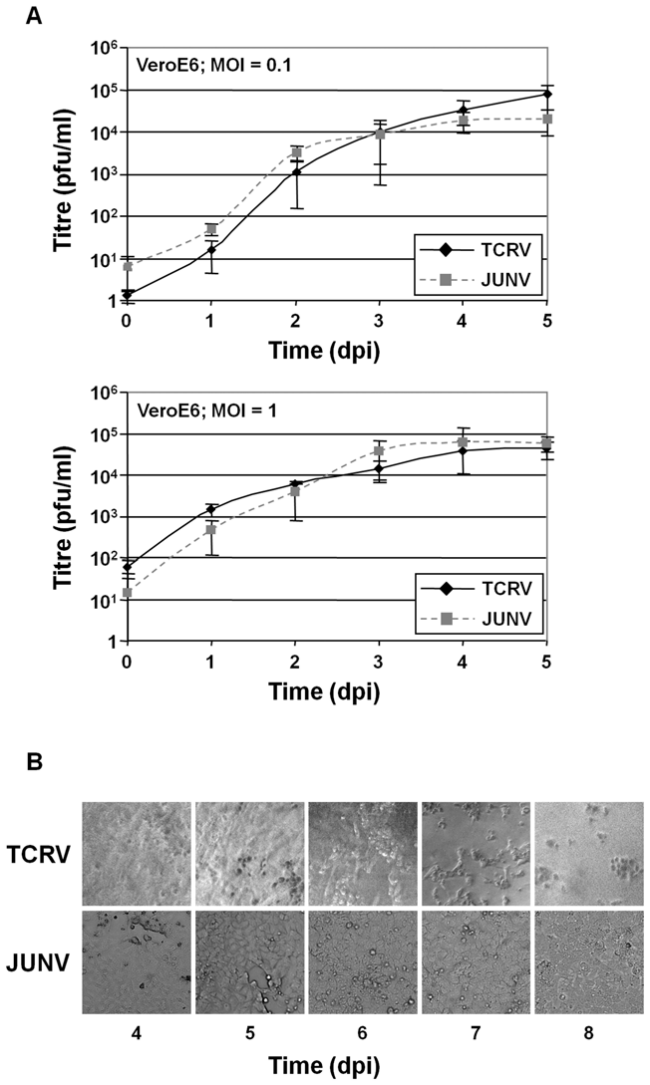
Comparison of virus growth and CPE formation during TCRV and JUNV infection of VeroE6 cells. (A) Virus growth during TCRV and JUNV infection. Sub-confluent VeroE6 cells were infected in a 6 well format with either TCRV or JUNV at an MOI of either 0.1 (upper panel) or 1 (lower panel). Cell culture supernatants were harvest immediately after infection and every 24 h thereafter for a period of 5 days. Virus titres in these supernatants were determined by plaque assay (B) CPE formation during TCRV and JUNV infection. VeroE6 cells were infected as described for (A) and CPE formation was monitored. Data are shown starting from the time of onset of CPE formation.

Before beginning our analyses in macrophages, we first compared the replication of TCRV and JUNV by examining the kinetics of virus growth for these two viruses in VeroE6 cells, which are known to be broadly permissive for arenavirus infection [24]. Cells were infected at MOIs of 1 or 0.1. However, regardless of the MOI used to establish the infection, no significant difference was observed between TCRV and JUNV during growth in VeroE6 cells (Fig. 1A). Further, neither virus produced a marked cytopathic effect (CPE) during the first 4 days of infection, during which time logarithmic virus growth occurred. However, at later time points TCRV produced a strong CPE and ultimately resulted in almost complete detachment of the monolayer (Fig. 1B). In contrast, for JUNV only limited cell rounding and in some cases the formation of small transient holes in the monolayer was observed at similar time points. These data indicate that under permissive conditions no fundamental defect exists that compromises the capacity of TCRV for replication, in comparison to JUNV.

**Figure 2:**
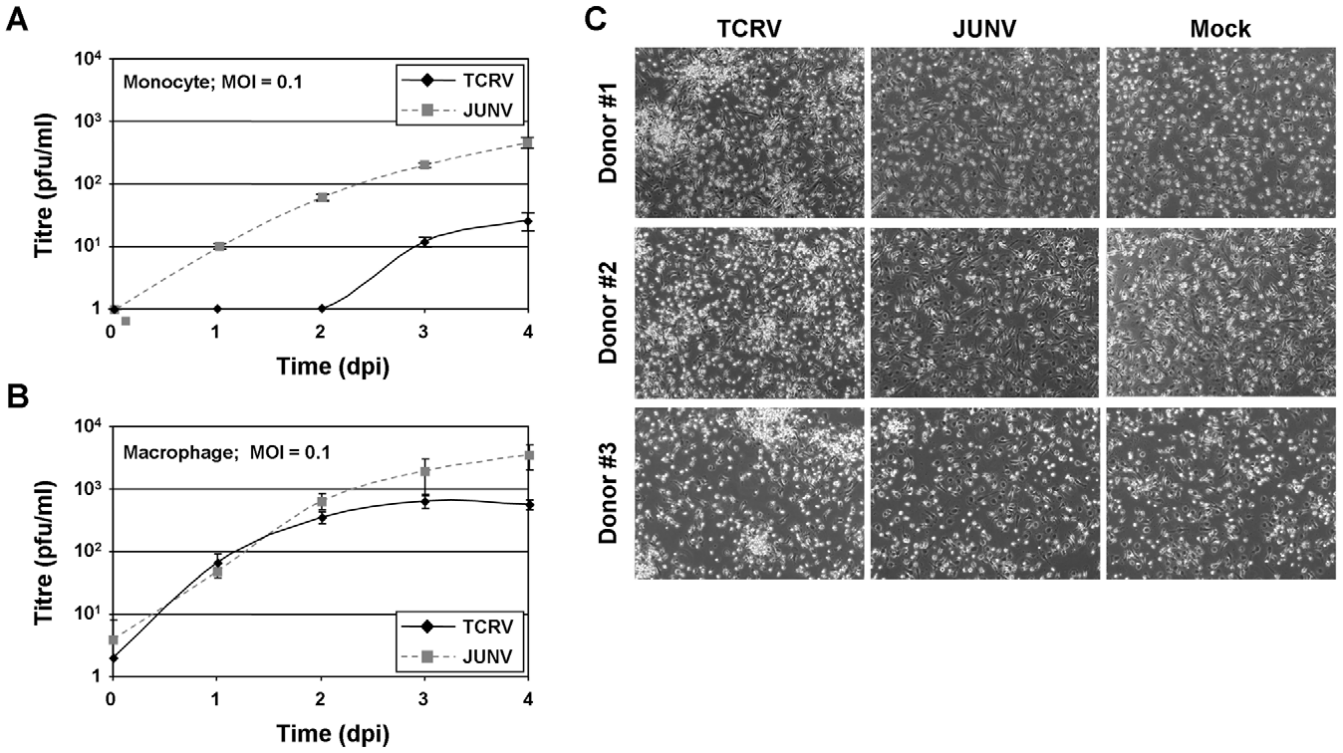
Comparison of virus titre and CPE production during infection of primary human monocytes and macrophages. Growth kinetics of TCRV and JUNV in (A) primary human monocytes and (B) primary human macrophages. Primary human monocytes and macrophages were isolated by adherence of peripheral blood mononuclear cell fractions in 6-well plates with subsequent maturation for either 1 (monocyte) or 7 (macrophage) days. Subsequently, monocyte (upper panel) and macrophage (lower panel) populations were infected at an MOI of 0.1 with either TCRV or JUNV. Cell culture supernatants were harvest immediately after infection and every 24 h thereafter for a period of 5 days. Virus titres in these supernatants were determined by plaque assay. (C) Cytopathic effect during infection of primary human monocytes with TCRV and JUNV. The cells from which supernatants were collected for analysis in (A) were analysed by light microscopy for the formation of cytopathic effect in comparison to mock infected cells 4 days after infection.

In contrast, infection of primary human monocytes and macrophages with JUNV at an MOI of 0.1 resulted in significantly faster growth and approximately 10-fold higher end-point titres compared to TCRV infection (Fig. 2A and 2B). It was also observed that, while growth of TCRV was reduced compared to JUNV in both cell types, differences during growth in macrophages were only present at later time points (days 3 and 4), while differences in monocytes were observed at all time points. Interestingly, while neither virus produced significant CPE during infection of macrophages (data not shown), a characteristic CPE was observed in monocytes during TCRV infection. In these samples we observed the formation of cell clusters throughout the culture (Fig. 2C), which resembled those reported for filovirus infected monocyte cultures [17], and was suggestive of activation.

### TCRV infection of primary monocytes and macrophages leads to production of IL-6, IL-10 and TNF-α

**Figure 3:**
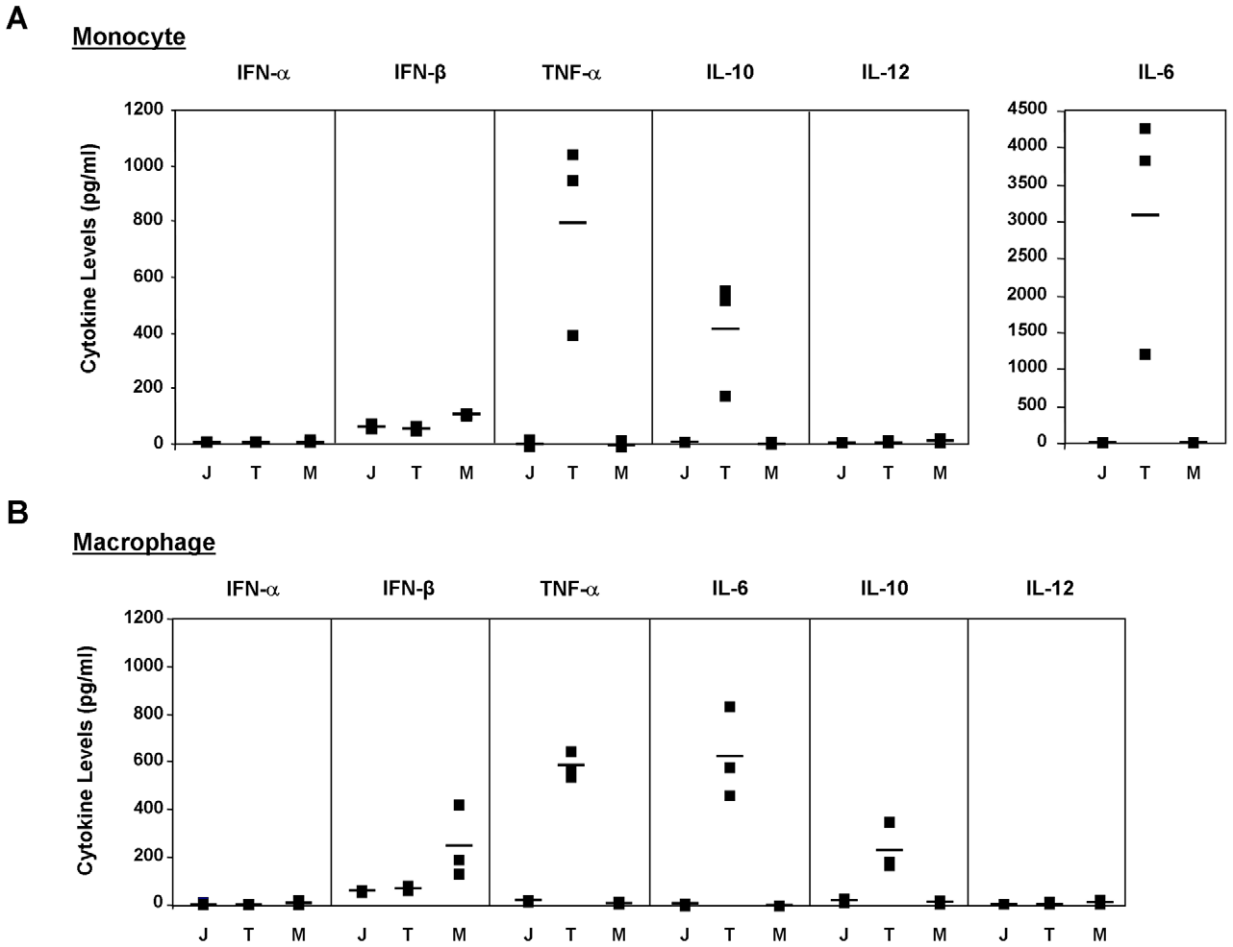
Cytokine production during TCRV and JUNV infection of primary human monocytes and macrophages. (A) Cytokine production during infection of primary human monocytes. Primary human monocytes were infected at an MOI of 0.1 with either TCRV or JUNV or mock infected in a 6-well plate with supernatants collected for cytokine analysis. Supernatants were analysed after 4 days using commercially available cytokine ELISA kits for IFN-α and IFN-β (Pestka Biomedical Laboratories) as well as IL-6, IL-10, IL-12 and TNF-α (R&D Systems) according to the manufacturer's instructions. The cytokine concentration for each of three donors (black box), as well as the mean for each group (black bar), is indicated for each treatment group: TCRV infected (T), JUNV infected (J) or Mock (M). (B) Cytokine production during infection of primary human macrophages. Primary human macrophages were infected and cytokine analysis performed on collected supernatants as described in (A).

**Figure 4:**
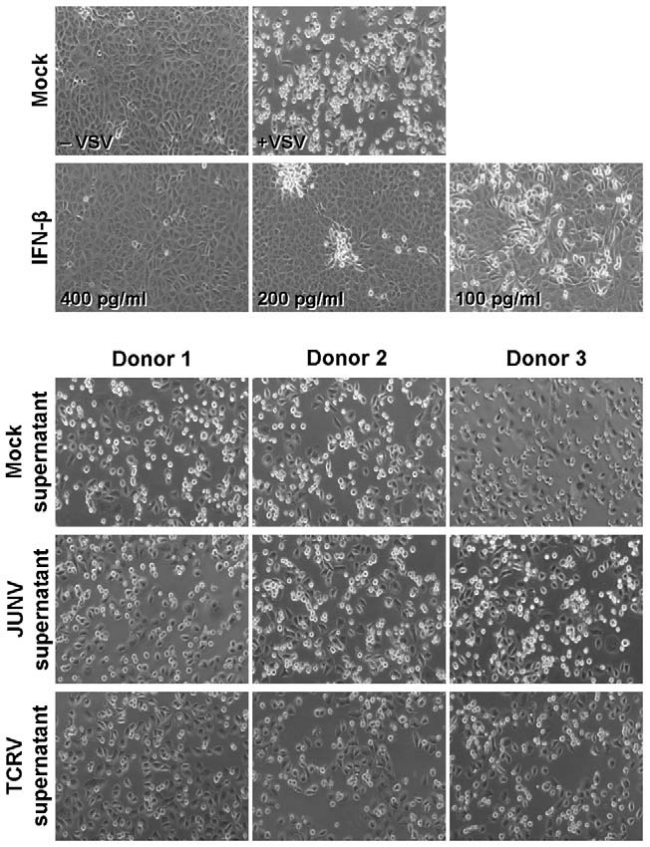
Bioassay for IFN production during TCRV and JUNV infection of primary human monocytes. IFN-α/β activity in monocyte culture supernatants was detected using a biological assay for protection against vesicular stomatitis virus (VSV) infection and its associated cytopathic effects. For this assay, VeroE6 cells cultured in 12-well plates were treated with supernatants from monocytes infected with either JUNV or TCRV, supernatants from mock infected monocytes or dilutions of a commercial recombinant human IFN-β standard (50 ng/ml to 100 pg/ml). After 24 h the cells were washed and infected with VSV at an MOI of 0.001. Cells were then incubated at 37°C for 18 h before being examined for the formation of CPE.

In order to clarify a potential role for macrophage-derived cytokines in the pathogenesis of New world arenavirus infections, we next examined the production of several key cytokines (IFN-α, IFN-β, TNFα, IL-6, IL-10 and IL-12) following infection of primary human monocyte and macrophage cultures with either TCRV or JUNV at an MOI of 0.1. Supernatants were harvested at day 4 post-infection and examined using commercial cytokine ELISAs. This time-point was consistent with the accumulation of maximum virus titres in the cultures and the end of productive virus growth for both viruses (Fig. 2A and 2B). Surprisingly, we observed that JUNV infection did not up-regulate any of the cytokines examined (Fig. 3). However, TCRV infection led to the release of significant amounts of TNFα, as well as IL-6 and IL-10 (Fig. 3). These levels constituted an up-regulation in comparison to both JUNV infected and mock infected cells. IL-12 production was not affected by JUNV or TCRV infection and, surprisingly, neither IFN-α nor IFN-β were up-regulated in either the monocyte or macrophage cultures (Fig. 3). This was despite the observation that both monocytes and macrophages prepared in this fashion were able to produce detectable levels of IFN-α and IFN-β in response to SeV infection, and IL-12 in response to LPS stimulation (data not shown). In order to confirm the absence of IFN-α and IFN-β in JUNV and TCRV infected supernatants, we further analysed them using an IFN bioassay. This assay is based on the ability of IFN-α and IFN-β to inhibit VSV infection of various cell types, which can then be clearly seen as a reduction in CPE formation and monolayer loss [25], [26]. Using this approach we could confirm that supernatants derived from JUNV, TCRV and mock-infected monocyte cultures did not contain significant amounts of IFN-α/β (Fig. 4). Further, based on comparison with standards containing known amounts of recombinant human IFN-β we could determine that all supernatant contained less than the equivalent of 100 pg/ml (27 IU) of IFN-β, which approached the background level of detection in the commercial ELISA assays used, thus confirming the absence of IFN in these samples.

### Infection is required for the production of cytokines in response to Tacaribe virus

**Figure 5:**
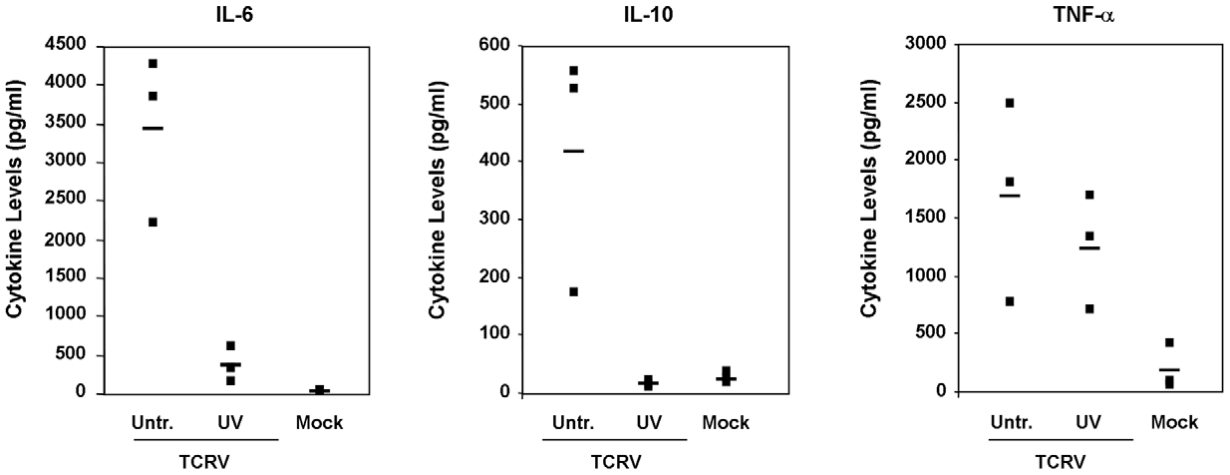
Cytokine production during exposure of primary human monocytes and macrophages to inactivated TCRV. Samples containing stock TCRV were inactivated by exposure to UV light for 60 min before being used to infect primary human monocytes at a dilution equivalent to an MOI of 0.1. Infection with untreated TCRV or mock infection of cells served as controls. Supernatants from the infected cells were analysed after 4 days using commercially available cytokine ELISA kits for IL-6 (left panel), IL-10 (center panel) and TNF-α (right panel) (R&D Systems) according to the manufacturer's instructions. The cytokine concentration for each of three donors (black box), as well as the mean for each group (black bar) is indicated for each treatment group: Untreated TCRV (Untr.), UV-inactivated TCRV (UV) or Mock (M).

In order to determine whether cytokine production by TCRV infected primary human monocytes and macrophages is dependent on productive infection of these cells, we next exposed fresh monocyte cultures to UV-inactivated TCRV or JUNV and compared cytokine production to that in cells infected with untreated virus. Monocyte cultures were chosen for this analysis as they showed the highest levels of cytokine production and, therefore, provided the most sensitive system for comparison of the cytokine response before and after virus inactivation. We first confirmed that UV-inactivation resulted in reduction of virus titres to less than 1 pfu/ml by analysing samples of inactivated virus stocks via plaque assay (data not shown), consistent with previous reports demonstrating the efficacy of this method [22], [23]. Supernatants from monocytes cultures infected with these inactivated virus samples were collected after 4 days and examined for production of TNFα, IL-6 and IL-10 by ELISA. UV-inactivated JUNV particles were unable to stimulate production of TNFα, IL-6 or IL-10 (data not shown). When cells were treated with UV-inactivated TCRV almost no release of IL-6 or IL-10 was detected, in contrast to infection with infectious TCRV (Fig. 5). Interestingly, when UV-inactivated TCRV was used TNF-α levels were not significantly reduced (Fig. 5). These observations then clearly indicate that the production of IL-6 and IL-10 during TCRV infection of monocytes is strongly dependent on productive virus infection, while TNF-α production is largely independent of viral replication. Further, this result demonstrates that IL-6 is not being induced as a by-product of TNF-α up-regulation in the monocyte cultures, but is separately and independently regulated by TCRV infection.

### Cytokine production in response to Tacaribe virus infection does not directly impair arenavirus infection in macrophages

**Figure 6:**
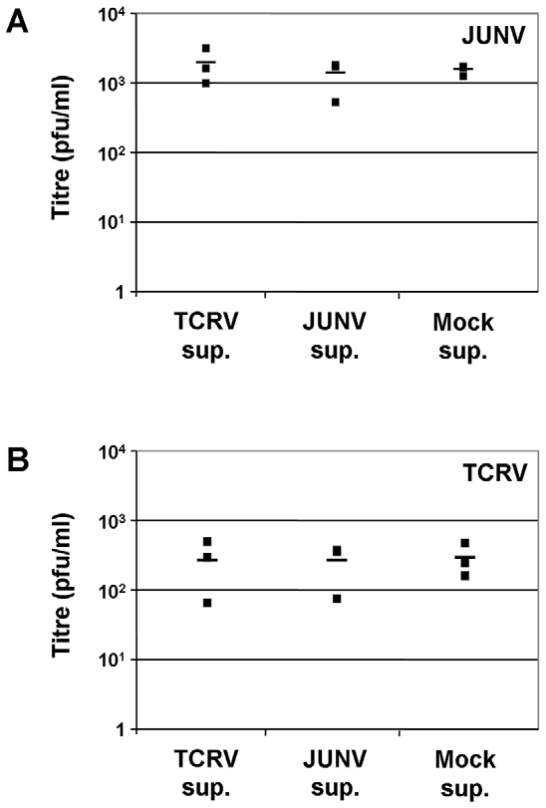
Comparison of virus titre during infection in the presence or absence of cytokine-containing supernatants. Cytokine supernatants collected from primary human monocytes infected at an MOI of 0.1 with either TCRV or JUNV or mock infected at 4 days post-infection were subjected to ultracentifugation to remove virus particles. Macrophage cultures were then pretreated with the clarified supernatant of TCRV, JUNV or mock infected monocytes for 2 h prior to the addition of known amounts of (A) JUNV or (B) TCRV for infection. Samples were collected after 4 days and plaque assays performed to determine virus titres.

Since TCRV infection in both monocytes and macrophages is somewhat impaired compared to infection with JUNV, we were interested to determine whether there is a direct effect of the cytokines produced during TCRV infection on arenavirus infection in these cell types. To assess this, we first removed virus particles from the TCRV culture supernatants by ultra-centrifugation. The complete removal of TCRV (residual titres <1 pfu/ml) using this method was verified by plaque assay (data not shown). These clarified supernatants were then used to pre-treat fresh macrophage cultures before JUNV or TCRV, corresponding to an MOI of 0.1, was added. Macrophage, rather than monocyte cultures were used because they generated higher virus titres during infection, and thus provided a more sensitive system to analyse any effect on virus growth. These experiments revealed no influence of the cytokines contained in TCRV-infected monocyte supernatants on the titres obtained following either TCRV or JUNV infection (Fig. 6). Thus the production of TNF-α, IL-6 and IL-10 during TCRV infection does not seem to directly influence virus replication in these cells.

Taken together, our data clearly demonstrate that, while pathogenic New World arenavirus infection is not associated with the production of cytokines in primary human monocytes or macrophages, infection with the non-pathogenic TCRV produced significant levels of IL-6, IL-10 and TNF-α. The production of these cytokines was further shown to be mechanistically distinct as both IL-6 and IL-10 production were dependent on productive virus infection while TNF-α production was also induced by UV-inactivated particles. Further, we found that TCRV has a reduced ability to replicate in these important primary target cells, although we could show that this was not directly attributable to the presence of the cytokines produced in response to infection.

## Discussion

The observation that the infection of macrophages with many different VHFs consistently induces an inflammatory cytokine response [27] has led to a general model for VHF pathogenesis in which excessive macrophage activation leads to induction of a cytokine storm. This systemic expression of elevated levels of cytokines is then proposed to result in the development of a septic shock-like syndrome, characterized by hypotension, insufficient tissue perfusion and multiple organ dysfunction [28], a disease profile that is consistent with what is known of fatal cases from many of the hemorrhagic fevers, including those caused by arenaviruses. While it has been shown that *in vitro* replication of LASV in macrophages occurs without inducing production of inflammatory cytokines [29], [30], the lack of coagulation disorders [27], as well as the low incidence of severe disease and corresponding low case fatality rates (1–2%), raise questions as to whether LASV can be considered as a representative VHF. In contrast, the New World Arenaviruses produce much more typical hemorrhagic fevers, characterized by severe disease with high case fatality rates (15–30%) and coagulation abnormalities. On this basis we considered that infection with these viruses may, therefore, provide a more suitable model for understanding arenavirus-induced hemorrhagic fever pathogenesis.

A significant role for macrophages in the pathophysiology of JUNV infection has been indicated by studies in animal models, where they have been shown to produce high titres of infectious virus [31], [32]. Further, macrophage maturity has been shown to be important for age-related resistance to JUNV infection in the rat model [33]. A role for macrophages in human JUNV infection is also suggested by observations of productive infection of patient peripheral blood mononuclear cells (PBMCs) during the acute phase of disease [34], as well as the increased levels of several cytokines (IFN-α, IL-6, IL-8, IL-10 and TNF-α) in patient serum that are known to be produced by, among others, macrophages [10], [11], [12]. However, a definitive link between JUNV infection of macrophages and cytokine production, as well as an understanding of the role these cytokines play in the development of disease is notably lacking. In order to address this topic we have examined the patterns of cytokine expression during infection of primary monocytes and macrophages with either JUNV or the closely related TCRV, which is apathogenic for humans. It could be shown that both viruses grow readily in these cells *in vitro*, although TCRV growth was decreased by ∼1 log compared to JUNV titres in both monocytes and macrophages. Since TCRV growth in a broadly permissive cell line (VeroE6) was comparable we suggest that this growth impairment is a feature of infection in monocytic cells, rather than being indicative of a general growth defect in TCRV. This reduction in infectious virus particle production in monocytic cells could play a potentially important role in limiting spread from these cells into additional target tissues. Such a role in dissemination of virus following their initial infection has been proposed to be an important function of infected macrophages in the context of filovirus infection [35], and is supported by kinetic analyses of filovirus infected non-human primates [36]. Further, we observed that, while the growth of TCRV is decreased in comparison to JUNV in both macrophages and monocytes, this difference is most prominent at early time points in monocytes, while in macrophages differences are only apparent at late time points. This suggests that the basis for inhibition of TCRV growth in these two cell types may be quite different and that cell attachment plays a direct role in the ability of monocytic cells to be infected by TCRV. Further, the early suppression of TCRV growth in monocytes speaks for the involvement of an early step in the infectious process. It is, therefore, tempting to speculate that monocytic cell attachment may affect the expression of the as yet unknown TCRV receptor and thereby influence the susceptibility of adherent versus non-adherent monocytic cells to TCRV infection. If this is indeed the case, similar effects on the *in vivo* susceptibility of circulating blood monocytes during infection could then affect the potential for subsequent systemic spread of the virus and thereby contribute to pathogenesis.

Surprisingly, despite the fact that several pro- and anti-inflammatory cytokines have been shown to be elevated in patients with severe and/or fatal JUNV infection [10], [11], [12], we could not identify any production of IFN-α, IFN-β, TNF-α, IL-6, IL-10 or IL-12 during *in vitro* infection of human monocytes or macrophages with JUNV. In contrast, infection with the human apathogenic TCRV resulted in a strong up-regulation of several of these cytokines (IL-6, TNF-α and IL-10) in a manner that, for IL-6 and IL-10, was dependent on productive virus infection. Interestingly, for TNF-αactivation was largely independent of productive infection, a finding that also indicates that induction of IL-6 and IL-10 are not being induced as a by-product of TNF-α up-regulation, but rather that these mediators are independently induced. Although the mechanism by which TNF-α induction occurs remains elusive, a similar phenomenon has been reported for a diverse range of viruses, including Ebola virus, Marburg virus, Herpes Simplex virus and Reovirus [17], [37], [38]. For Ebola virus, infection-independent TNF-α activation has been suggested to occur in response to receptor cross-linking by the viral surface glycoproteins [39] and, based on our observations, we can propose that a similar process may take place with arenavirus particles as well. Considering the similar morphologies demonstrated by the different arenaviruses, the lack of TNF-α activation with JUNV UV-inactivated particles might be related of the differing receptor usage between TCRV and JUNV [40].

While our findings initially appear to be in contrast with the established cytokine storm model of viral hemorrhagic fever pathogenesis, as well as JUNV patient data, which clearly shows up-regulation of numerous cytokines, we believe that these findings can be reconciled. In filovirus patients it was observed that a strong but transient release of cytokines early in infection is linked to patient survival [41], [42], while terminal patients develop some of the same cytokines but only late in infection [41]. Thus, it appears not to be the production of proinflammatory cytokines as such that is devastating for the infected patient, but rather their uncoordinated release. In further support of such a model it has been shown that macrophages infected *in vitro* with LASV also support virus replication, but without activation or induction of cytokine responses, while infection with the closely related but apparently human apathogenic Mopeia virus results in up-regulation of mRNAs encoding IFN-α, IFN-β, TNF α and IL-6 [29], [30], [43]. Taken together with our data, this seems to suggest that strong cytokine activation during macrophage infection is a feature of apathogenic arenavirus infection, regardless of whether the virus in question is an Old World or New World arenavirus. Further, an ability to suppress cytokine activation during macrophage infection may be equally typical of pathogenic family members. Thus our findings seem to be in agreement with an increasing body of evidence suggesting that an early induction of cytokines and the corresponding innate immune responses can play a protective role in preventing the development of hemorrhagic fever during arenavirus infection.

The absence of IFN-α during JUNV infection of monocytes and macrophages was particularly unexpected, given the very high levels of IFN-α known to be present in infected patients. This finding clearly suggests that the IFN-α found during infection is of non-monocytic origin and is rather derived from other leukocyte populations. In particular, the potential of dendritic cell populations as sources of interferon during infection will have to be closely examined. The lack of IFN production during JUNV infection can also potentially be explained by the existence of two known viral IFN antagonists, NP and Z [44], [45]. However, the absence of appreciable IFN-α or IFN-β production during TCRV infection was surprising. This finding suggests that, at least in monocytic cells, TCRV is capable of circumventing the type-I interferon response. This is despite evidence indicating that TCRV NP is impaired in its ability to inhibit IFN induction, compared to other arenaviruses NPs [44]. While JUNV Z has recently been suggested to serve as an additional interferon antagonist for JUNV [45], there is currently no information regarding a similar function for TCRV Z. Thus, it remains possible that this or other viral proteins may have as yet undescribed interferon antagonistic activities. Alternatively, it has also been suggested that TCRV may be able to avoid triggering the interferon response during infection by means of an additional non-coded terminal nucleotide in the virus genome, which is added during replication [46] and may mask the 5′ tri-phosphate RNA end that serves as a trigger for activation of the interferon cascade through RIG-I [47]. Together with previous reports showing that the nucleoprotein of Pichinde virus, another non-human pathogenic New World arenavirus, acts as an efficient interferon antagonist [44], our own findings support the position that evasion of the interferon response is necessary for virus survival in both pathogenic and apathogenic viruses, rather than being an inherent indicator of pathogenic potential.

One possibility that was examined in this study was that the high levels of cytokine production in monocytes and macrophages during TCRV infection might play a direct role in restricting virus growth in these cells. However, an analysis of the growth of both JUNV and TCRV in macrophages pre-treated with TCRV-derived cytokine-containing supernatant showed no influence of these cytokines on the virus titres obtained following infection. This observation indicates that any protective role of these cytokines is unlikely to be due to a direct role in limiting TCRV replication in macrophages. However, they may still play a systemic role in regulation of the immune response to TCRV infection. In light of the mounting evidence showing a role of macrophage-derived cytokine expression in apathogenic arenavirus infection, we propose that up-regulation of IL-6 and TNF-α in the context of TCRV infection appropriately activates the acute phase response rather than contributing to immunopathology. In contrast, the absence of IL-6 and TNF-α production after infection with JUNV might allow the virus to continue to replicate unchecked and thereby achieve the high virus loads and broad tissue dissemination that are indicative of many viral hemorrhagic fevers [27]. The up-regulation of IL-10 by TCRV is also intriguing since this cytokine is known to play a role in the activation and maturation of B-cells. Given the role for the antibody response in the control of New World arenavirus infection, as is clearly demonstrated by the success of passive antibody transfer in the treatment of AHF patients [9], its up-regulation may also be of particular significance for preventing the development of disease during human TCRV infection, While antibody production, and particularly the production of neutralizing antibody, does occur during JUNV infection, we would suggest that the induction of IL-10 following TCRV infection of monocytes and macrophages may be critical for inducing a stronger and/or more rapid humoral immune response to infection, which could aid in rapid virus clearance and thus prevent the onset of clinical disease.

Taken together, this study shows that, unlike the better understood filovirus hemorrhagic fevers, pathogenic New World arenavirus infection is associated with a lack of cytokine up-regulation during *in vitro* macrophage infection, despite productive infection. In contrast, infection with the human apathogenic TCRV was associated with high levels of IL-6, IL-10 and TNF-α. In conjunction with the observations from severe clinical AHF cases, which show high levels of these same cytokines in symptomatic JUNV patients, this finding suggests a more complex relationship between cytokine production during infection of primary target cells (i.e. macrophages) and the subsequent development of disease, and provides much needed insight into the immune processes necessary for the successful control of arenavirus infection.
